# Perforation of the Intestine in Typhoid Fever

**Published:** 1905-03-18

**Authors:** 


### PERFORATION OF THE INTESTINE IN TYPHOID FEVER.

Most people will agree with Dr. Basile[1] that it is fair to rate the mortality after perforation in typhoid fever at 100 per cent. in the absence of operative interference. If this premiss be accepted it is idle to waste words upon reputed barriers to operation, such as the multiplicity of the lesions and the exhaustion of the patient, for, as the author says, there is no reason in depriving a patient of his only present means of cure because he still runs the risk of a problematical further perforation in the future; moreover, actual experience in cases where operation has been successful shows that second perforations are rare.

The burden of the communication under discussion is the necessity for identifying the accident at the earliest possible moment after its occurrence; and the justice of the attitude is self-evident, though the difficulty of the identification need not be emphasised. This difficulty depends to a large extent on the fact that many patients, by the time that perforation occurs, are in a stage of the malady so advanced that the ordinary symptoms of rupture of a hollow viscus (which present themselves so dramatically, for instance, in a sudden perforation of a gastric ulcer) are veiled by exhaustion. But it may be fairly said that the diagnosis is not of so high an importance under such conditions, for a patient so debilitated as to lack response upon perforation is already moribund, and intervention useless.

In dealing with the early diagnosis of rupture the author passes in review the classical indices of the lesion. The pulse, for instance, is small, frequent, and thready; but so it often is apart from perforation. Again, it is affirmed that such a pulse associated with a marked drop in temperature is strong evidence for perforation. While admitting this, Dr. Basile asserts that this conjunction is often absent in the vital early stages; moreover, it is quite as likely to mean hæmorrhage. Meteorism, loss of liver dulness, vomiting, and hiccough are all intrinsically untrustworthy, though valuable as confirmatory evidence. Similarly a sudden leucocytosis may occur without perforation, and, *per contra*, in the early hours of perforation there may be no leucocytosis. The three paramount symptoms are abdominal pain, if sudden and severe; abdominal rigidity, and an abdominal facies. When these three symptoms coincide, as they generally do, the diagnosis is clinched.

Dealing with the possibility of error in the diagnosis Dr. Basile says, "Better a laparotomy without a perforation than a perforation without a laparotomy," and bases his motto on several cases in which operation performed for the relief of a rupture of the gut, which in fact did not exist, was followed by no harmful result to the patient. Such an unnecessary operation is of course to be deplored, but there is no doubt that in skilled hands an exploratory operation is harmless enough to be fully justified, considering the grave possibilities latent in a doubtful instance.

The case for early operation is backed by the following statistics:—Cazin, 27·6 per cent. of successes; Keen, 23 per cent.; Harte, 26 per cent. The author further reports seven cases from his own observation, of whom five recovered, a percentage of 71·4 of successes. Even this extraordinary recovery rate appears to represent the facts unfairly, for one of the patients who ultimately died survived the operation for upwards of a week, and was found at the autopsy to be the subject of an abscess in the spleen, and of a resolving pneumonia. Of these seven cases one was of the ambulatory type. In six of the seven the perforation took place during the initial illness, while in one it occurred in the course of a relapse.

While we cannot but suspect that the author has been unusually lucky in his experience, figures far less successful would amply justify insistence on the necessity of early surgical treatment for an accident almost inevitably fatal in the ordinary course of nature.

[1] Il Policlinico, Feb. 5 and 12, 1905.

